# Prenatal plasma concentrations of Perfluoroalkyl and polyfluoroalkyl substances and neuropsychological development in children at four years of age

**DOI:** 10.1186/s12940-019-0493-3

**Published:** 2019-06-13

**Authors:** Jinbo Niu, Hong Liang, Youping Tian, Wei Yuan, Hong Xiao, Hui Hu, Xiaowei Sun, Xiuxia Song, Sheng Wen, Li Yang, Yanfeng Ren, Maohua Miao

**Affiliations:** 1 The First People’s Hospital of Jianshan, Jiaxing, Zhejiang Province China; 2 NHC Key Lab of Reproduction Regulation (Shanghai Institute of Planned Parenthood Research), Fudan University, Shanghai, China; 3 Department of Pharmaceutical Outcomes & Policy, College of Pharmacy, University of Florida, 1225 Center Drive, HPNP 3338, Gainesville, FL 32610 USA; 4 Department of Epidemiology, College of Public Health and Health Professions and College of Medicine, University of Florida, 2004 Mowry Road, Gainesville, FL 32610 USA; 5 National Reference Laboratory of Dioxin, Institute of Health Inspection and Detection, Hubei Provincial Academy of Preventive Medicine, Hubei Provincial Center for Disease Control and Prevention, Wuhan, 430079 China; 6 Department of Public Educaion, Weifang Medical University, 7166 Baotong west Road, Weifang, 261053 Shandong Province China; 7 Department of Health Statistics, School of Public Health and Management, Weifang Medical University, 7166 Baotong west Road, Weifang, 261053 Shandong Province China

**Keywords:** Perfluoroalkyl and polyfluoroalkyl substances, Neuropsychology, Age and stage questionnaire, Prenatal concentrations

## Abstract

**Objective:**

Perfluoroalkyl and polyfluoroalkyl substances (PFASs) are persistent pollutants and have endocrine disruptive and neurotoxic effects. The association between maternal PFAS concentrations and neuropsychological development in children is inconclusive. The present study aimed to examine the effect of maternal PFAS concentrations on neuropsychological development in 4-years-old children.

**Methods:**

We used data from Shanghai-Minhang Birth Cohort, which recruited pregnant women at 12–16 gestational weeks. Among 981 women having PFAS measurement, 533 mother-child pairs were included in the study. A total of eight PFASs were measured, including perfluorooctane sulfonate (PFOS), perfluorooctanoic acid (PFOA), perfluorohexane sulfonate (PFHxS), perfluorononanoic acid (PFNA), perfluorodecanoic acid (PFDA), perfluoroundecanoic acid (PFUdA), perfluorododecanoic acid (PFDoA), and perfluorotridecanoic acid (PFTrDA). When infants turned 4 years old, mothers were asked to complete the Ages and Stages Questionnaires® (ASQ) to assess neuropsychological development of their children. Poisson regression model with robust variance estimates was used to examine the association between maternal PFAS concentrations and each developmental subscale of the ASQ.

**Results:**

Prenatal plasma concentrations of most PFASs tended to be associated with increased risk of development problem in personal-social skills, including PFHxS, PFOS, PFOA, PFNA, PFDA, and PDUdA, and the associations for PFNA and PFDA were significant (per natural log unit increase: RR_PFNA_ = 1.92, 95% CI: 1.21, 3.05; RR _PFDA_ = 1.66, 95% CI: 1.17, 2.37). In stratified analyses by child’ sex, the consistent pattern of higher risk of developmental problems in personal-social skills associated with most PFASs was mainly observed among girls (RR_PFOS_ = 2.56, 95% CI: 1.20, 5.45; RR_PFOA_ = 9.00, 95% CI: 3.82, 21.21; RR_PFNA_ = 3.11, 95% CI: 1.36, 7.13; RR_PFDA_ = 2.20, 95% CI: 1.21, 4.00; RR_PFUdA_ = 2.44, 95% CI: 1.14, 5.20; RR_PFDoA_ = 1.62, 95% CI: 1.04, 2.54). Boys with higher maternal PFOA concentrations had a decreased risk of developmental problems in gross motor skills (RR = 0.47, 95% CI: 0.25, 0.89).

**Conclusion:**

Prenatal plasma PFAS concentrations were associated with neuropsychological development in girls at 4 years of age, mainly in the subset of personal-social skills.

**Electronic supplementary material:**

The online version of this article (10.1186/s12940-019-0493-3) contains supplementary material, which is available to authorized users.

## Introduction

Perfluoroalkyl and polyfluoroalkyl substances (PFASs), a group of synthetic chemicals with hydrophobic (water-repelling) and oleophobic (oil-repelling) properties, have been extensively used in many consumer products, including oil, stain, grease, and water-repellent coatings on carpet, textiles, leather, and paper [1]. PFASs are bio-accumulative and have a long elimination half-life of 2–9 years [2]. Thus, they have been detected in wildlife and humans worldwide [3]. Concerns about the health/toxic effects of PFASs, particularly for in-utero exposure, have been raised for decades [4].

In animal studies, maternal PFAS exposure during pregnancy was associated with somatic growth, e.g., birth weight and size [5]. Moreover, maternal PFAS exposure can induce alteration in neuropsychological development of fetuses and neonates. Deranged spontaneous behavior was observed in adult mice following perfluorooctane sulfonate (PFOS) and perfluorooctanoic acid (PFOA) exposure during neonatal period, manifesting in hyperactivity and irreversibly reduced habituation [6]. In addition, maternal PFOS or PFOA exposure during pregnancy can lead to decreased motor function and delayed learning in rat offspring [7].

The associations between maternal PFAS exposure and neuropsychological development in children have also been examined in human studies, but the findings are inconsistent [8–11]. A prospective birth cohort study in Japan showed an association between prenatal PFOA exposure and mental developmental in girls aged 6 months, but not in those aged 18 months [12]. The biopersistent organochlorines in diet and human fertility (INUENDO) cohort study reported that prenatal exposure to PFOS and PFOA may have adverse effects on children’s neurobehavioral development, specifically in terms of hyperactive behavior [13]. However, in the Danish National Birth Cohort (DNBC) established between 1996 and 2002, maternal serum levels of PFOA and PFOS were not associated with behavioral and motor coordination problems in 7-year old children [10]. A nested case-control study from the DNBC suggested that prenatal PFAS exposure did not increase the risk of attention-deficit/hyperactivity disorder (ADHD) or autism in children [11]. In a 2005–2006 cohort study in the Mid-Ohio Valley, children in the highest quartile of maternal PFOA concentrations had higher intelligence quotient scores and decreased ADHD scores at ages 6–12 years compared with those in the lowest quartile [8].

Previous studies examining the associations between maternal PFAS exposure and child neurodevelopment focused on PFOS and PFOA. However, other commonly used PFAS compounds, such as perfluorohexane sulfonate (PFHxS), perfluorononanoic acid (PFNA), and perfluorodecanoic acid (PFDA), can be detected in more than 85% of individuals in China and in France [14, 15] and 50% of individuals in USA [16] and are less studied. Maternal concentrations of PFASs, including PFOS, PFOA PFHxS, PFNA, and PFDA, are much higher in the Shanghai area than in the United States, Europe and Asian countries [14]. In the present study, we used data from the Shanghai-Minhang Birth Cohort Study (S-MBCS) to examine associations between maternal concentrations of eight PFASs and the neurodevelopment of 4-year-old children assessed by the Ages and Stages Questionnaires, 3rd edition® (ASQ − 3).

## Methods

### Participants

The Shanghai-Minhang Birth Cohort was established between April and December 2012 [14, 17]. While attending routine antenatal examinations at the Maternal and Child Health Hospital of Minhang district in Shanghai, all pregnant women at 12–16 weeks of gestation was invited to participate in the study. Inclusion criteria included: being registered residents of Shanghai, having no history of chronic disease of the liver, kidney, or other organs, planning to deliver in this study hospital, and willingness to participate in specified interviews during pregnancy and after delivery.

In total, 1292 eligible pregnant women completed a structured questionnaire, among them, 981 provided a fasting blood sample at enrollment. Sixty-seven eligible pregnant women were excluded due to referral to other hospitals (*n* = 28), twin pregnancy (*n* = 8), and abortion or stillbirth (*n* = 31). The remaining 1225 women delivered singleton live births. Structured questionnaires were administered postnatally during home visits at 4 years of age to collect information on the child’s physical and neuropsychological development. ASQ-3 included in the structured questionnaire was used to identify children at a risk for neuropsychological developmental delay. We obtained complete ASQ-3 assessment data from 661 participants at 4 years of age. The present study included 533 mother-infant pairs who had measurements of prenatal PFAS concentration and child neuropsychological development at 4 years of age.

### Exposure assessment

Maternal blood samples were collected at enrollment. After separating plasma from whole blood, the plasma samples were stored at − 80 °C until shipment using dry ice to the Center for Disease Control and Prevention in Hubei Province for quantitative analyses of 11 PFASs.

Eleven PFASs in each plasma sample were measured using high-performance liquid chromatography coupled with tandem mass spectrometry (Agilent Technologies Inc., USA). Detailed information on sample preparation, separation, quantification, quality control, and limit of detection (LOD) has been described previously [14]. The following eight PFASs with detection rates ≥90% were included in the present study for statistical analyses: PFHxS, PFOS, PFOA, PFNA, PFDA, perfluoroundecanoic acid (PFUdA), perfluorododecanoic acid (PFDoA), and perfluorotridecanoic acid (PFTrDA).

### Assessment of children’s neuropsychological development

ASQ-3, contains 30 items designed to assess infant neuropsychological development in children aged 1–60 months. It covers five developmental subscales: communication, gross motor function, fine motor function, problem-solving ability, and personal-social skills [18]. A detailed description of Chinese translation, training, and process of validation of ASQ-3 has been described elsewhere [19]. The Simplified Chinese version of ASQ-3 has good internal consistency (Cronbach’s α = 0.80), high test-retest reliability (correlation coefficient = 0.8), and high validity (sensitivity = 87.50%, specificity = 84.48%) [19].

At home visits, parents or other caregivers were asked whether the child performs the described behavior based on three alternatives: “yes” (10 points), “sometimes” (5 points) and “not yet” (0 points). The score of each ASQ-3 subscale was highly skewed, with few children scoring lower than 2 standard deviations (SDs) below the standardized mean. Thus, the 10th percentile score of each subscale was used to identify children at a potential risk of developmental problem/delay, i.e., if scores on any subscale were less than or equal to the 10th percentile, the child was classified as having a developmental problem/delay. Additional file 1: Table S1 presents the rates of potential developmental problem assessed at 4 years of age in the study.

### Covariates and potential confounders

Trained interviewers used a structured questionnaire at enrollment to collect information on maternal age, education, height, pre-pregnancy weight, parity, health status, per capita household income, and lifestyle. Body mass index (BMI) was calculated as body weight in kilograms divided by squared body height in meters. The information on child’s sex and gestational age was extracted from the study hospital’s medical records. Potential confounders were identified based on previous literature of potential determinants of early childhood development, available data in the present study, and results of bivariate analyses examining the relationship with neuropsychological problems (*P* < 0.20). Maternal age at enrollment (years), pre-pregnancy BMI (kg/m^2^), parity, per capita household income (< 4000, 4000–8000, and > 8000 CNY/month), passive smoker (yes/no), gestational age (weeks), and child’s sex (Boy/Girl) were included as covariates in the final model.

### Statistical analysis

We first described and compared the demographic characteristics of included and excluded mother-infant pairs. The distributions of prenatal plasma concentrations of PFASs were presented by geometric means (GMs), SD, and percentiles. Risk ratios (RRs) and associated 95% confidence intervals (CIs) were estimated for the association between each PFAS and each developmental subscale of ASQ using Poisson regression analysis with robust variance estimates [20]. Prenatal PFAS concentrations were natural log (ln) transformed to approximate a normal distribution for regression analysis and were treated as continuous independent variables in all models. Values below the LOD were replaced with LOD/√2.

Considering that a previous study reported the sex-specific effect of PFASs on neurobehavioral problems [21], we introduced a cross product term for child’s sex with each individual PFAS to evaluate potential interaction effects. As several interaction items showed statistical significance (*P* < 0.10), we performed stratified analyses by child’s sex. PFAS concentrations were also categorized into tertiles according to its distribution in the subjects and were analyzed using Poisson regression. The lowest tertile was used as the reference group.

Additionally, we fit generalized additive models to investigate a potential nonlinear relationships between maternal PFAS concentration and neurobehavioral problems, and visually inspected plots of the smoothed data. Several associations between ln-transformed PFASs and children’s neuropsychological development showed nonlinear (Additional file 1: Figure S1-S5). In order to make our results comparable to other studies and considering that the aim of our study was inference rather than prediction, we still primarily presented the results of linear models (PFAS concentrations as continuous and categorical variables, respectively),

The Statistical Analysis System (SAS, version 9.3; SAS Institute, Inc., Cary, NC, USA) was used for statistical analysis. *P* values < 0.05 from two-tailed tests were considered statistically significant.

## Results

Table 1 presents characteristics of mother-child pairs included and excluded in the study. The mean age of the included mothers at recruitment was 27.9 years, and 18.7 and 5.9% of them were underweight and overweight, respectively. Included parents tended to have high educational attainment, with 78.0% having college or above degrees. Among included mothers, the majority were nulliparous and had household income per capita > 4000 CNY/month, and over 40% of them were exposed to secondhand smoke during pregnancy, while only 0.87% of them reported alcohol consumption during pregnancy. About 18.6% of included mothers had depressive symptoms during pregnancy. The mean gestational weeks of included mothers at birth of children was 39.5 weeks and 55.7% of children were boys. The characteristics of excluded mother-pairs were comparable with those included, except that the proportion of nulliparous women was slightly higher among the included than those excluded (86.9% vs. 82.7%).

**Table 1:** Characteristics of included and excluded mother-child pairs

Characteristics	Included (*N* = 533) n (%) / Mean ± SD	Excluded (*N* = 692) n (%) / Mean ± SD	*P*-value of Student’s t-test or Chi-square test
Maternal age at enrollment (years)			
Mean ± SD	27.9 ± 3.4	27.8 ± 3.4	0.7451
< 25	81 (15.2)	103 (14.9)	0.7900
25–30	299 (56.1)	404 (58.4)	
≥ 30	153 (28.7)	185 (26.7)	
Maternal pre-pregnancy BMI (kg/m^2^)			
Mean ± SD	20.6 ± 2.4	20.4 ± 2.4	0.1905
< 18.5	98 (18.7)	146 (21.5)	0.2112
18.5–24.9	395 (75.4)	506 (74.4)	
≥ 25	31 (5.9)	28 (4.1)	
Maternal education			
Blow high school	41 (7.7)	78 (11.3)	0.1064
High School	76 (14.3)	99 (14.3)	
College or above	415 (78.0)	514 (74.4)	
Paternal education			
Blow high school	28 (5.25)	57 (8.28)	0.9430
High School	76 (14.26)	119 (17.3)	
College or above	429 (80.49)	512 (74.42)	
Per capita household income (CNY)			
< 4000	113 (21.3)	140 (20.6)	0.0282
4000–8000	212 (40.0)	277 (40.8)	
> 8000	205 (38.7)	262 (38.6)	
Parity			
Nulliparous	459 (86.9)	569 (82.7)	0.0432
Multiparous	69 (13.1)	119 (17.3)	
Maternal alcohol consumption during pregnancy			
No	454 (99.13)	508 (98.64)	0.4743
Yes	4 (0.87)	7 (1.36)	
Maternal passive smoking before conception			
No	307 (57.8)	420 (60.9)	0.2811
Yes	224 (42.2)	270 (39.1)	
Maternal prenatal depressive symptoms			
No	434 (81.43)	551 (79.62)	0.4309
Yes	99 (18.57)	141 (20.38)	
Sex of child			
Boy	297 (55.7)	370 (53.7)	0.4816
Girl	236 (44.3)	319 (46.3)	
Gestational age (weeks)			
Mean ± SD	39.5 ± 1.3	39.51 ± 1.5	0.6182
< 37	19 (3.6)	26 (3.8)	0.8640
≥ 37	513 (96.4)	666 (96.2)	

Missing data: Included: pre-pregnancy BMI (*n* = 9), maternal education (*n* = 1), per capita household income (*n* = 3), parity (*n* = 5), maternal alcohol consumption during pregnancy (*n* = 75) and maternal passive smoking before conception (*n* = 2); Excluded: pre-pregnancy BMI (*n* = 12), maternal education (*n* = 1), paternal education (*n* = 4), per capita household income (*n* = 13), parity (*n* = 4), maternal alcohol consumption during pregnancy (*n* = 177), maternal passive smoking before conception (*n* = 2), and maternal prenatal depressive symptoms (*n* = 1)

PFHxS, PFOS, PFOA, PFNA, PFDA, PFUdA, and PFDoA were detected in all maternal plasma samples, while PFTrDA was detected in 90.6% of samples. PFOA had the highest exposure levels (GM = 20.0 ng/ml), followed by PFOS (GM = 10.8 ng/ml). The concentrations of PFHxS, PFDA, PFNA, and PFUdA were one order of magnitude lower than those of PFOA and PFOS, while those of PFDoA and PFTrDA were two orders of magnitude lower (Table 2). Prenatal PFAS concentrations were similarly distributed in girls and boys (Additional file 1: Table S2).

**Table 2:** Maternal PFASs concentrations (ng/mL) at 12–16 gestational weeks (*N* = 533) in Shanghai, China

PFAS	LOD	>LOD (N %)	GM (GSD)	Percentiles				
				5th	25th	50th	75th	95th
PFHxS	0.015	533 (100)	2.7 (1.6)	1.4	2.1	2.8	3.5	5.7
PFOS	0.02	533 (100)	10.8 (1.8)	4.5	7.6	10.8	15.8	25.2
PFOA	0.01	533 (100)	20.0 (1.6)	9.3	15.3	19.9	27.4	38.9
PFNA	0.02	533 (100)	1.8 (1.6)	0.8	1.3	1.8	2.5	3.9
PFDA	0.01	533 (100)	2.1 (1.9)	0.7	1.4	2.1	3.2	6.3
PFUdA	0.01	533 (100)	1.6 (1.9)	0.5	1.0	1.6	2.5	4.4
PFDoA	0.015	533 (100)	0.1 (2.9)	LOD	0.1	0.1	0.2	0.4
PFTrDA	0.02	483 (90.6)	0.1 (2.9)	LOD	0.1	0.1	0.2	0.4

*LOD* Limit of detection, *GM* Geometric mean, *GSD* Geometric standard deviation

**Fig. 1:**
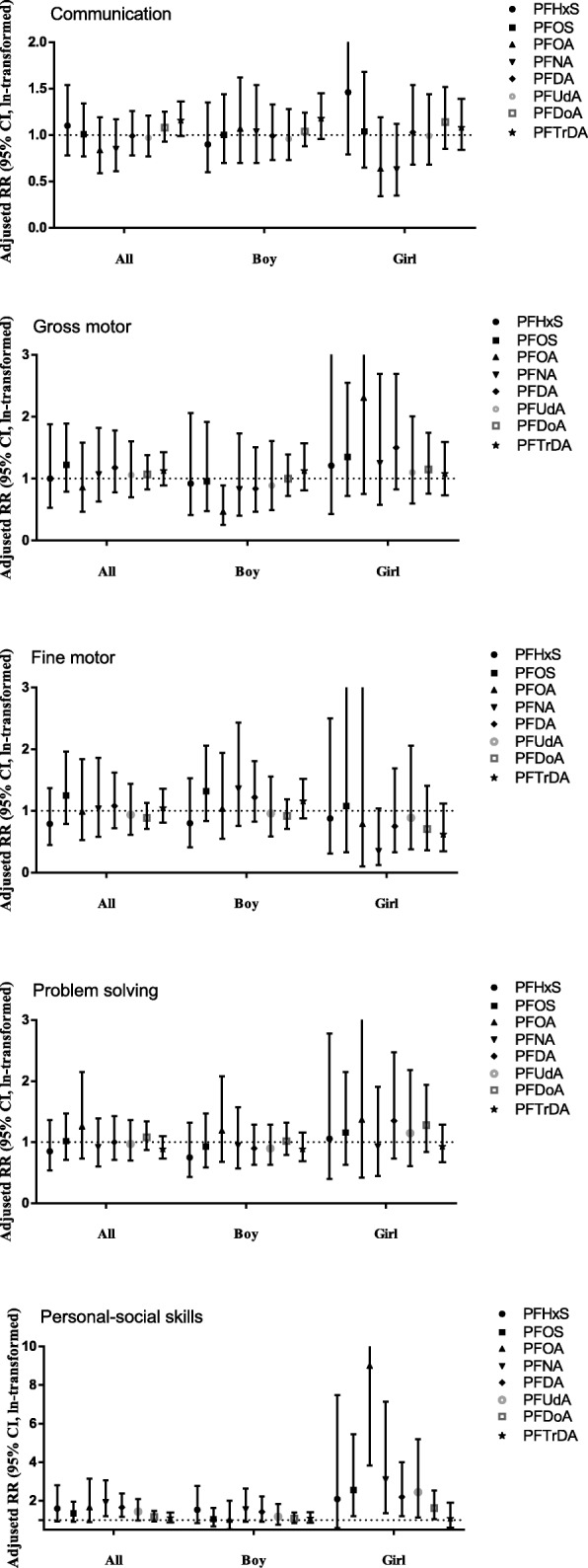
Associations between maternal PFAS concentrations (ln-transformed) and neuropsychological problems of ASQ scales at 4 years of age in Poisson regression with robust variance estimates

Table 3 and Fig. 1 present associations between maternal ln-transformed PFAS concentrations (as continuous variables) and children’s neurodevelopmental problems at 4 years of age. There was a pattern of higher risks of developmental problem in personal-social skills associated with higher prenatal plasma concentrations of PFHxS, PFOS, PFOA, PFNA, PFDA, and PDUdA, with significant associations for PFNA and PFDA (per natural log unit increase: RR_PFNA_ = 1.92, 95% CI: 1.21, 3.05; RR _PFDA_ = 1.66, 95% CI: 1.17, 2.37). Children with higher prenatal PFTrDA concentrations tended to have a slightly higher risk of developmental problem in communication, with borderline significance (RR = 1.16 for per natural log unit increase, 95% CI: 0.99, 1.36), but the association was not observed for other PFASs. No statistically significant association was observed between maternal PFAS concentrations and developmental problems in gross motor, fine motor, and problem solving skills.

**Table 3:** Associations between maternal PFAS concentrations (ln-transformed) and neuropsychological problems of ASQ scales at 4 years of age in Poisson regression with robust variance estimates

PFAS	Communication		Gross motor		Fine motor		Problem solving		Personal–social skills	
	CRR (95% CI)	ARR (95% CI)	CRR (95% CI)	ARR (95% CI)	CRR (95% CI)	ARR (95% CI)	CRR (95% CI)	ARR (95% CI)	CRR (95% CI)	ARR (95% CI)
PFHxS	1.15 (0.84, 1.58)	1.10 (0.78, 1.54)	1.07 (0.56, 2.03)	1.00 (0.53, 1.88)	0.80 (0.44, 1.49)	0.79 (0.45, 1.37)	0.88 (0.57, 1.35)	0.85 (0.54, 1.36)	1.49 (0.82, 2.71)	1.60 (0.92, 2.80)^#^
PFOS	0.99 (0.76, 1.28)	1.01 (0.77, 1.34)	1.16 (0.76, 1.76)	1.22 (0.79, 1.89)	1.20 (0.82, 1.77)	1.25 (0.79, 1.96)	1.05 (0.76, 1.44)	1.02 (0.71, 1.47)	1.23 (0.83, 1.82)	1.34 (0.91, 1.96)
PFOA	0.83 (0.59, 1.15)	0.84 (0.59, 1.19)	0.85 (0.46, 1.55)	0.86 (0.47, 1.58)	0.93 (0.51, 1.70)	0.99 (0.53, 1.84)	1.22 (0.73, 2.03)	1.26 (0.73, 2.15)	1.43 (0.77, 2.64)	1.67 (0.89, 3.14)
PFNA	0.83 (0.61, 1.13)	0.85 (0.61, 1.17)	1.03 (0.62, 1.72)	1.07 (0.63, 1.82)	0.97 (0.55, 1.70)	1.04 (0.58, 1.86)	0.94 (0.63, 1.38)	0.92 (0.60, 1.39)	1.50 (0.93, 2.40)	1.92 (1.21, 3.05)^*^
PFDA	0.97 (0.77, 1.21)	0.99 (0.78, 1.26)	1.17 (0.78, 1.74)	1.18 (0.78, 1.78)	1.03 (0.72, 1.47)	1.08 (0.72, 1.62)	1.05 (0.77, 1.45)	1.00 (0.71, 1.43)	1.42 (1.00, 2.01)^*^	1.66 (1.17, 2.37)^*^
PFUdA	0.96 (0.77, 1.18)	0.97 (0.77, 1.21)	1.07 (0.71, 1.60)	1.06 (0.70, 1.60)	0.92 (0.61, 1.39)	0.94 (0.61, 1.44)	1.00 (0.73, 1.36)	0.97 (0.70, 1.36)	1.28 (0.89, 1.82)	1.44 (0.99, 2.09)
PFDoA	1.04 (0.91, 1.2)	1.08 (0.93, 1.25)	1.09 (0.85, 1.41)	1.07 (0.83, 1.38)	0.88 (0.70, 1.11)	0.89 (0.71, 1.13)	1.11 (0.90, 1.37)	1.08 (0.87, 1.34)	1.15 (0.92, 1.43)	1.17 (0.93, 1.47)
PFTrDA	1.10 (0.95, 1.27)	1.16 (0.99, 1.36)^#^	1.13 (0.91, 1.41)	1.13 (0.89, 1.43)	1.03 (0.82, 1.29)	1.05 (0.81, 1.36)	0.89 (0.74, 1.08)	0.89 (0.73, 1.10)	1.07 (0.86, 1.35)	1.11 (0.88, 1.38)

Models were adjusted for maternal age at enrollment (age), pre-pregnancy BMI (kg/m^2^), maternal education, paternal education, parity, per capita household income (< 4000, 4000–8000, and > 8000 CNY), maternal passive smoking (yes or no), maternal prenatal depressive symptoms (yes or no), gestational age (weeks), and child’s sex

*CRR* Crude risk ratio, *ARR* Adjusted risk ratio, *CI* Confidence interval

^#^ Marginally significant differences (0.05 < *p* < 0.10)

^*^ Statistically significant differences (*p* < 0.05)

When evaluating the potential effect modifications by child’s sex, some interaction items of maternal PFAS concentrations and child’s sex were statistically significant (*p* < 0.10) in the models examining PFOA and gross motor, PFNA and fine motor, and PFOS/PFOA and personal-social skills (Additional file 1: Table S3). The consistent pattern of higher risk of developmental problems in personal-social skills associated with most PFASs shown in Table 3 was mainly observed among girls (Table 4). Among these associations, those for PFOS, PFOA, PFNA, PFDA, PFUdA, and PFDoA concentrations were statistically significant (per natural log unit increase of PFAS concentrations: RR_PFOS_ = 2.56, 95% CI: 1.20, 5.45; RR_PFOA_ = 9.00, 95% CI: 3.82, 21.21; RR_PFNA_ = 3.11, 95% CI: 1.36, 7.13; RR_PFDA_ = 2.20, 95% CI: 1.21, 4.00; RR_PFUdA_ = 2.44, 95% CI: 1.14, 5.20; RR_PFDoA_ = 1.62, 95% CI: 1.04, 2.54). There were no significant associations between PFAS concentrations and problems in other subscales in girls (Table 4). No clear association between PFAS concentrations and problems in each subscale was observed in boys (Table 4). Only boys with higher prenatal PFOA concentrations had a decreased risk of developmental problems in gross motor skills (per natural log unit increase: RR = 0.47, 95% CI: 0.25, 0.89) (Table 4).

**Table 4:** Associations between maternal PFAS concentrations (ln-transformed) and neuropsychological problems of ASQ scales at 4 years of age among boys and girls in Poisson regression with robust variance estimates

PFAS	Communication ARR (95% CI)		Gross motor ARR (95% CI)		Fine motor ARR (95% CI)		Problem solving ARR (95% CI)		Personal–social skills ARR (95% CI)	
	Girl (*N* = 236)	Boy (*N* = 297)	Girl (*N* = 236)	Boy (*N* = 297)	Girl (*N* = 236)	Boy (*N* = 297)	Girl (*N* = 236)	Boy (*N* = 297)	Girl (*N* = 236)	Boy (*N* = 297)
PFHxS	1.46 (0.79, 2.70)	0.90 (0.60, 1.35)	1.21 (0.43, 3.41)	0.92 (0.41, 2.06)	0.88 (0.31, 2.50)	0.80 (0.41, 1.53)	1.06 (0.40, 2.78)	0.75 (0.43, 1.32)	2.09 (0.58, 7.49)	1.53 (0.84, 2.78)
PFOS	1.04 (0.65, 1.68)	1.00 (0.70, 1.44)	1.35 (0.72, 2.55)	0.96 (0.48, 1.92)	1.08 (0.33, 3.59)	1.32 (0.84, 2.06)	1.16 (0.63, 2.15)	0.93 (0.59, 1.47)	2.56 (1.20, 5.45)^*^	1.05 (0.67, 1.64)
PFOA	0.64 (0.34, 1.19)	1.07 (0.70, 1.62)	2.31 (0.75, 7.10)	0.47 (0.25, 0.89)^*^	0.79 (0.10, 6.29)	1.04 (0.55, 1.94)	1.37 (0.42, 4.47)	1.19 (0.68, 2.08)	9.00 (3.82, 21.21)^*a^	1.03 (0.53, 2.01)
PFNA	0.63 (0.35, 1.12)	1.04 (0.70, 1.54)	1.25 (0.58, 2.69)	0.83 (0.40, 1.73)	0.35 (0.12, 1.04)	1.36 (0.76, 2.43)	0.93 (0.45, 1.91)	0.95 (0.57, 1.57)	3.11 (1.36, 7.13)^*^	1.55 (0.91, 2.64)
PFDA	1.03 (0.68, 1.54)	0.99 (0.73, 1.33)	1.50 (0.83, 2.69)	0.84 (0.47, 1.51)	0.75 (0.33, 1.69)	1.22 (0.83, 1.81)	1.35 (0.73, 2.47)	0.84 (0.55, 1.27)	2.20 (1.21, 4.00)^*^	1.42 (0.91, 2.22)
PFUdA	0.99 (0.68, 1.44)	0.96 (0.73, 1.28)	1.10 (0.60, 2.01)	0.89 (0.49, 1.61)	0.89 (0.38, 2.06)	0.96 (0.59, 1.56)	1.15 (0.61, 2.18)	0.90 (0.63, 1.29)	2.44 (1.14, 5.20)^*^	1.18 (0.75, 1.84)
PFDoA	1.14 (0.85, 1.52)	1.04 (0.88, 1.24)	1.15 (0.76, 1.74)	1.00 (0.72, 1.39)	0.71 (0.36, 1.41)	0.92 (0.71, 1.19)	1.28 (0.84, 1.94)	1.02 (0.79, 1.32)	1.62 (1.04, 2.54) ^*^	1.08 (0.84, 1.38)
PFTrDA	1.08 (0.84, 1.39)	1.18 (0.96, 1.45)	1.08 (0.73, 1.59)	1.13 (0.81, 1.57)	0.62 (0.35, 1.12)	1.16 (0.88, 1.52)	0.93 (0.67, 1.29)	0.89 (0.69, 1.16)	1.08 (0.61, 1.90)	1.10 (0.86, 1.41)

Models were adjusted for maternal age at enrollment (ages), pre-pregnancy BMI (kg/m^2^), maternal education, paternal education, parity, per capita household income (< 4000, 4000–8000, and > 8000 CNY), maternal passive smoking (yes or no), maternal prenatal depressive symptoms (yes or no), and gestational age (weeks)

^a^The strong association may be caused by statistical imprecision mainly due to the imbalanced distribution of numbers of children having potential problems by PFOA concentrations. As shown in Table 5, there was no cases in the lowest tertile group of PFOA concentrations among girls, which potentially influenced the estimation in Poisson regression model

*ARR* Adjusted risk ratio, *CI* Confidence interval

^*^ Statistically significant differences (*p* < 0.05)

We further examined the associations between maternal PFAS concentration as categorical variables and developmental problems by child’s sex (Table 5). Generally, the models using categorized PFAS variables showed similar results as reported in the main analyses. In girls, there was a consistent pattern of increased risk of problems in personal-social skills associated with higher maternal PFAS concentrations except PFTrDA, although the estimates became imprecise. For PFOA, the regression model did not converge because there was no child with developmental problem in the lowest tertile group. A linear trend was observed between tertiles of PFOS, PFNA, PFDA, and PFDoA and problems in the subscale (P for trend =0.0027, 0.0417, 0.0110, and 0.0159, respectively, Table 5). In addition, prenatal PFNA concentrations were associated with a decreased risk of communication problems; adjusted RRs were 0.73 (95%CI: 0.41, 1.32) for the middle tertile and 0.50 (95% CI: 0.26, 0.94) for the highest tertile (P for trend =0.0292). There were no clear associations between maternal PFAS concentrations and problems in each subscale among boys. However, boys with higher maternal PFTrDA concentrations were more likely to have communication problem; the adjusted RRs was 1.53 (95%CI: 0.92, 2.55) for middle tertile and 1.83 (95%CI: 1.08, 3.12) for the highest tertile (P for trend = 0.0218). Boys in the middle tertile of maternal PFUdA concentrations had more problems in fine motor scale (RR = 2.19, 95%CI: 1.16, 4.17) (Table 5).

**Table 5:** Associations between maternal PFAS concentrations (divided by tertiles) and neuropsychological problems of ASQ scales at 4 years of age among boys and girls in Poisson regression with robust variance estimates

PFAS	Communication ARR (95% CI)		Gross motor ARR (95% CI)		Fine motor ARR (95% CI)		Problem solving ARR (95% CI)		Personal–social skills ARR (95% CI)	
	Girl (*N* = 236)	Boy (*N* = 297)	Girl (*N* = 236)	Boy (*N* = 297)	Girl (*N* = 236)	Boy (*N* = 297)	Girl (*N* = 236)	Boy (*N* = 297)	Girl (*N* = 236)	Boy (*N* = 297)
PFHxS										
Low tertile	Ref	Ref	Ref	Ref	Ref	Ref	Ref	Ref	Ref	Ref☆
Middle tertile	0.99 (0.53, 1.83)	0.64 (0.39, 1.05)	1.55 (0.68, 3.55)	0.76 (0.35, 1.67)	1.46 (0.35, 6.10)	0.55 (0.27, 1.13)	0.81 (0.35, 1.89)	0.85 (0.44, 1.64)	1.62 (0.43, 6.08)	0.68 (0.30, 1.55)
High tertile	1.24 (0.65, 2.36)	0.90 (0.59, 1.36)	1.43 (0.55, 3.68)	0.57 (0.26, 1.24)	0.50 (0.05, 5.00)	0.73 (0.40, 1.36)	1.24 (0.48, 3.21)	0.61 (0.32, 1.18)	2.56 (0.73, 9.03)	1.77 (0.94, 3.34)
PFOS										
Low tertile	Ref	Ref	Ref	Ref	Ref	Ref	Ref	Ref	Ref☆	Ref
Middle tertile	0.52 (0.26, 1.04)#	1.16 (0.76, 1.77)	0.81 (0.27, 2.43)	0.94 (0.43, 2.07)	0.79 (0.13, 4.91)	1.87 (0.92, 3.80)#	0.55 (0.15, 2.07)	1.21 (0.65, 2.28)	0.32 (0.04, 2.77)	1.47 (0.76, 2.84)
High tertile	1.10 (0.63, 1.92)	0.89 (0.53, 1.51)	1.61 (0.62, 4.16)	0.91 (0.37, 2.24)	1.98 (0.34, 11.53)	1.19 (0.52, 2.71)	2.00 (0.77, 5.17)	0.66 (0.29, 1.48)	2.97 (0.90, 9.84)#	1.18 (0.57, 2.44)
PFOA										
Low tertile	Ref☆	Ref	Ref	Ref☆	Ref	Ref	Ref	Ref	Ref	Ref
Middle tertile	0.86 (0.49, 1.50)	1.02 (0.65, 1.6)	1.08 (0.33, 3.57)	0.51 (0.23, 1.11)#	0.50 (0.13, 1.93)	0.88 (0.43, 1.80)	0.93 (0.35, 2.46)	1.09 (0.56, 2.12)	not converge	1.60 (0.80, 3.19)
High tertile	0.55 (0.28, 1.10)#	0.96 (0.61, 1.52)	1.90 (0.66, 5.44)	0.45 (0.19, 1.04)#	1.32 (0.29, 5.93)	0.91 (0.47, 1.78)	0.90 (0.36, 2.26)	1.10 (0.58, 2.06)	not converge	1.50 (0.77, 2.93)
PFNA										
Low tertile	Ref★	Ref	Ref	Ref	Ref	Ref	Ref	Ref	Ref★	Ref
Middle tertile	0.73 (0.41, 1.32)	1.19 (0.76, 1.88)	0.96 (0.29, 3.20)	0.91 (0.39, 2.08)	1.15 (0.25, 5.24)	1.72 (0.82, 3.62)	0.56 (0.20, 1.55)	1.24 (0.64, 2.43)	2.87 (0.37, 22.20)	1.23 (0.62, 2.47)
High tertile	0.50 (0.26, 0.94)^*^	1.17 (0.72, 1.91)	1.50 (0.54, 4.21)	1.06 (0.48, 2.35)	0.46 (0.10, 2.11)	1.72 (0.82, 3.59)	0.93 (0.4, 2.17)	1.08 (0.52, 2.25)	5.68 (0.82, 39.34)	1.70 (0.86, 3.36)
PFDA										
Low tertile	Ref	Ref	Ref	Ref	Ref	Ref	Ref	Ref	Ref★	Ref
Middle tertile	0.61 (0.31, 1.20)	1.01 (0.66, 1.55)	1.16 (0.36, 3.71)	0.87 (0.42, 1.81)	2.04 (0.43, 9.66)	1.65 (0.87, 3.13)	0.50 (0.14, 1.74)	1.27 (0.69, 2.33)	2.16 (0.26, 17.69)	1.14 (0.57, 2.28)
High tertile	1.02 (0.54, 1.91)	0.88 (0.55, 1.42)	1.83 (0.62, 5.39)	0.53 (0.21, 1.32)	0.40 (0.03, 4.74)	0.80 (0.37, 1.76)	1.71 (0.70, 4.15)	0.65 (0.31, 1.36)	8.68 (1.41, 53.43)*	1.52 (0.74, 3.09)
PFUdA										
Low tertile	Ref	Ref	Ref	Ref	Ref	Ref	Ref	Ref	Ref☆	Ref
Middle tertile	0.64 (0.32, 1.25)	1.18 (0.78, 1.80)	0.44 (0.12, 1.53)	1.14 (0.54, 2.43)	2.10 (0.43, 10.41)	2.19 (1.16, 4.17)*	0.27 (0.05, 1.33)	1.57 (0.81, 3.03)	0.79 (0.11, 5.56)	1.77 (0.90, 3.47)#
High tertile	0.93 (0.52, 1.66)	0.83 (0.50, 1.38)	1.29 (0.53, 3.16)	0.77 (0.31, 1.92)	1.35 (0.18, 10.02)	0.64 (0.26, 1.56)	1.35 (0.62, 2.94)	0.88 (0.40, 1.90)	3.72 (0.79, 17.62)^#^	1.12 (0.48, 2.63)
PFDoA										
Low tertile	Ref	Ref	Ref	Ref	Ref	Ref	Ref	Ref	Ref★	Ref
Middle tertile	0.78 (0.38, 1.58)	1.29 (0.82, 2.04)	0.81 (0.27, 2.38)	0.88 (0.41, 1.91)	0.48 (0.11, 2.06)	1.34 (0.70, 2.56)	1.25 (0.35, 4.41)	1.06 (0.54, 2.06)	3.99 (0.46, 34.3)	0.91 (0.47, 1.77)
High tertile	1.33 (0.72, 2.46)	1.14 (0.72, 1.82)	1.27 (0.51, 3.19)	0.93 (0.41, 2.09)	0.40 (0.07, 2.37)	0.84 (0.41, 1.75)	1.78 (0.61, 5.23)	1.13 (0.58, 2.18)	6.89 (0.97, 48.67)^#^	0.91 (0.46, 1.79)
PFTrDA										
Low tertile	Ref	Ref’	Ref	Ref	Ref	Ref	Ref	Ref	Ref	Ref
Middle tertile	1.19 (0.60, 2.36)	1.53 (0.92, 2.55)	0.94 (0.33, 2.66)	2.25 (0.82, 6.14)	0.44 (0.10, 1.98)	1.90 (0.86, 4.20)	1.69 (0.64, 4.47)	1.46 (0.75, 2.84)	0.79 (0.21, 2.96)	1.70 (0.85, 3.4)
High tertile	1.36 (0.69, 2.69)	1.83 (1.08, 3.12)*	0.85 (0.31, 2.29)	1.68 (0.56, 5.1)	0.28 (0.05, 1.61)	1.81 (0.78, 4.19)	0.88 (0.31, 2.52)	0.71 (0.29, 1.73)	0.84 (0.23, 3.03)	1.29 (0.61, 2.75)

Models were adjusted for maternal age at enrollment (ages), pre-pregnancy BMI (kg/m^2^), maternal education, paternal education, parity, per capita household income (< 4000, 4000–8000, and > 8000 CNY), maternal passive smoking (yes or no), maternal prenatal depressive symptoms (yes or no), and gestational age (weeks)

*ARR* Adjusted risk ratio, *CI* Confidence interval

^#^ Marginally significant differences (0.05 < *p* < 0.10)

^*^ Statistically significant differences (*p* < 0.05)

^★^*p*-trend < 0.05

^☆^0.05 < *p*-trend < 0.10

## Discussion

We found that girls with higher maternal concentrations of PFHxS, PFOS, PFOA, PFNA, PFDA, PFUdA, and PFDoA tended to have more problems in personal-social skills, while there was little evidence for consistent associations in boys.

In the study, median concentrations for maternal PFOS and PFOA were 10.8 and 19.9 ng/ml, respectively, which are one of the highest levels reported among pregnant women during the similar period (around 2012) compared to those of the studies conducted in the US (2.4 and 1.1 ng/ml) [16], Canada (4.6 and 1.7 ng/ml) [22], Denmark (8.23 and 2.0 ng/ml) [23], Australia (1.99 ad 0.86 ng/ml) [24], and Japan (3.52 and 1.27 ng/ml) [25], especially for PFOA. However, compared to the studies on prenatal PFAS concentrations and child’s neurodevelopment where the samples were collected mainly around 2000, PFOS concentrations were lower than most of the studies (ranged from 13.2 to 34.4 ng/ml) [10, 26] except the Hokkaido study in Japan [12, 27], while PFOA concentrations were much higher (ranged from 1.2 to 5.6 ng/ml in previous studies) [12, 28]. Different from other studies where PFOS has the highest concentrations, PFOA was the most predominant compound in the present study, which was also observed in another cohort study in Shanghai [29]. This may be explained by the findings that PFOA is the most prevalent compound in the surface water of Shanghai, accounting for 51–86% of total PFAS concentrations [30].

Results from epidemiological studies on neurodevelopmental impact of in utero PFAS exposure are inconsistent [10, 28, 31, 32]. A cohort study from Taiwan showed that prenatal exposure to PFOS might affect child neurodevelopment, especially gross motor development at 2 years of age [32]. Among 432 mother-daughter pairs from the Avon Longitudinal cohort, a 1 ng/mL increase in PFOS was associated with a 3.82-point (95% CI: − 6.18, − 1.47) lower vocabulary score at 15 months and a 0.80-point (95% CI: − 1.74, 0.14) lower language score at 38 months in daughters of mothers aged < 25 years [33]. A birth cohort study between 2002 and 2005 suggested an association between prenatal PFOA exposure and neurodevelopmental delay in 6-month-old females, as measured by mental scales of the second edition of the Bayley Scales of Infant Development [12]. In Oulhote et al.’s study, cross-sectional analyses at 7 years of age showed possible sex-dimorphic associations between PFAS concentrations and the Strengths and Difficulties Questionnaire (SDQ) scores; girls had consistently positive associations with SDQ scores, whereas boys exhibited a pattern of negative or null associations [9]. However, in a prospective study from the DNBC, maternal plasma levels of PFOA or PFOS were neither associated with mental developmental nor fine and gross motor developmental milestones in infants [28], and there was no association between PFAS concentration and behavioral and motor coordination problems at 7 years of age [10]. In a Norwegian birth cohort study, PFOA or PFOS measured in breast milk was not associated with child neuropsychological development assessed by ASQ at 12 and 24 months [31]. Moreover, behavioral development assessed by the Infant-Toddler Symptom Checklist (ITSC) found no consistent increase in behavioral problems at 12 and 24 months [31]. The inconsistent findings between the current study and previous studies may be due to differences in screening tools, children’s ages at assessment, and PFAS compounds measured and their concentrations [10, 28, 31].

The mechanism of the effect of PFASs on neurobehavioral development remains unclear. In animal studies, some PFASs may affect the cholinergic or dopaminergic system, resulting in altered responses to nicotine or imbalanced expression of the acetylcholine/dopamine phenotype [6]. PFASs also affect synaptogenesis and functional protein levels during neuron growth [34]. PFOA and PFOS significantly increased the levels of synaptophysin and tau in the cerebral cortex and hippocampus. Because these proteins are important for normal brain development, altered levels during a critical period of brain growth spurts could be one of the mechanisms of behavioral defects [34]. Other possible mechanisms include the endocrine-disrupting properties of PFASs in glucocorticoid, sex hormone [27] and thyroid hormone balance [35, 36]. Prenatal and postnatal exposure to PFASs interferes with thyroid hormone balance in humans, resulting in higher thyroid-stimulating hormone, decreased total/free triiodothyronine, and decreased total/free thyroxine levels [37–39], which may play a role in how PFASs affect human neurodevelopment.

Additionally, we found a consistent pattern of adverse effects on personal-social skills in girls but not in boys following prenatal PFAS exposure. Although chance findings may be incompletely excluded, mechanism investigations are warranted to understand the sex-specific association. In previous studies, prenatal PFOA exposure was associated with decreased Mental Developmental indices scores of female infants at 6 months of age [12], and PFOS exposure was associated with poorer metacognition scores only among school-aged girls [26]. These findings are in line with our results on personal-social skills which evaluate children’s abilities of self-helping and interacting with others. Human studies suggested that prenatal PFOA/PFOS exposure was significantly associated with testosterone/estradiol in male infants, progesterone levels, glucocorticoid levels, and DHEA levels in cord blood samples of both sexes [27, 40]. In addition, effects of PFAS exposure on thyroid hormone homeostasis may differ across sexes [41]. The hormonal effects of PFAS may differently affect the neurobehavioral development of males or females. However, whether the sex-specific difference is inherited due to sex or due to hormonal effects of PFAS exposure warrants further investigation.

One strength of our study is that the prospective design provides strong causality between PFAS exposure levels and child neurodevelopment. We measured two of the most frequently detected PFASs, PFOS and PFOA, as well as other PFAS compounds to provide a profile of the effects of commonly detected PFAS compounds. However, some potential limitations of the current study should be mentioned. First, there was considerable loss to follow-up for neurodevelopmental assessment during the study period, which increased the potential for selection bias. However, the characteristics of subjects in the original cohort were similar to those in the final sample in terms of maternal age, pre-pregnancy BMI, parity, and gestational age. Thus, the loss to follow-up was less likely to lead to substantial bias. Second, parental intelligence quotient (IQ) may affect the children’s ASQ score, however, the information about parental IQ has not been collected in the study. We adjusted for parental education in the models, which may partially control for the confounding effects of parental IQ. In addition, the relationship between PFASs and ASQ measures may have been confounded by postnatal environmental risk factors [42, 43]. The confounding effect of uncollected factors, e.g., child sleepiness and maternal self-regulation, cannot be adjusted for. Third, multiple comparisons may also be of concern because we examined the associations between eight PFASs and five subscales. However, for our main findings on the associations between PFAS concentrations and personal-social skills among girls, patterns were consistent across PFAS compounds, which were less likely to be due to chance alone.

## Conclusions

Maternal PFAS concentrations during pregnancy were inversely associated with neuropsychological development assessed by ASQ in 4-year-old children. Further investigation of the underlying mechanism of the effect of prenatal PFAS exposure on neuropsychological development is needed.

## Additional file

Additional file 1: **Table S1.** The rates of potential developmental problem assessed at 4 years of age by the Age and Stage Questionnaire (ASQ). **Table S2.** Maternal PFASs concentrations (ng/mL) at 12–16 gestational weeks stratified by child sex. **Table S3.** *P*-values of interaction item (PFASs*child sex) in the associations between maternal PFASs concentrations and child’s neurobehavioral problems assessed by ASQ at 4 years of age^*^. **Figure S1.** Adjusted generalized additive model plots of ln-transformed PFAS concentrations with developmental problems in communication among offspring. **Figure S2.** Adjusted generalized additive model plots of ln-transformed PFAS concentrations with offspring developmental problems in Gross motor among offspring. **Figure S3.** Adjusted generalized additive model plots of ln-transformed PFAS concentrations with developmental problems in Fine motor among offspring. **Figure S4.** Adjusted generalized additive model plots of ln-transformed PFAS concentrations with developmental problems in Problem solving among offspring. **Figure S5.** Adjusted generalized additive model plots of ln-transformed PFAS concentrations with developmental problems in Personal-social skills among offspring. (DOCX 15944 kb)

## Data Availability

The datasets used in the current study are available from the corresponding authors on reasonable request.
