# Supplementary material for: Prenatal plasma concentrations of Perfluoroalkyl and polyfluoroalkyl substances and neuropsychological development in children at four years of age
Source: Environ Health. 2019 Jun 13;18:53. doi: 10.1186/s12940-019-0493-3 (PMC6567504; doi:10.1186/s12940-019-0493-3)
Supplement: Supplementary file 1 — Table S1. The rates of potential developmental problem assessed at 4 years of age by the Age and Stage Questionnaire (ASQ). Table S2. Maternal PFASs concentrations (ng/mL) at 12–16 gestational weeks stratified by child sex. Table S3. P-values of interaction item (PFASs*child sex) in the associations between maternal PFASs concentrations and child’s neurobehavioral problems assessed by ASQ at 4 years of age*. Figure S1. Adjusted generalized additive model plots of ln-transformed PFAS concentrations with developmental problems in communication among offspring. Figure S2. Adjusted generalized additive model plots of ln-transformed PFAS concentrations with offspring developmental problems in Gross motor among offspring. Figure S3. Adjusted generalized additive model plots of ln-transformed PFAS concentrations with developmental problems in Fine motor among offspring. Figure S4. Adjusted generalized additive model plots of ln-transformed PFAS concentrations with developmental problems in Problem solving among offspring. Figure S5. Adjusted generalized additive model plots of ln-transformed PFAS concentrations with developmental problems in Personal-social skills among offspring. (DOCX 15944 kb) [file 12940_2019_493_MOESM1_ESM.docx]

**Additional file 1: Table S1** The rates of potential developmental problem assessed at 4 years of age by the Age and Stage Questionnaire (ASQ)

| Subscale | Rates of potential developmental problem (N=533)* | |
| --- | --- | --- |
|  | n | % |
| Communication | 133 | 24.95 |
| Gross motor | 54 | 10.13 |
| Fine motor | 54 | 10.13 |
| Problem solving | 72 | 13.51 |
| Personal–social skills | 56 | 10.51 |

^*^Definition: if a child’s score in each subscale was less than or equal to 10^th^ percentile of scores of the corresponding subscale, the child was defined as having potential developmental problem/delay.

**Table S2** Maternal PFASs concentrations (ng/mL) at 12-16 gestational weeks stratified by child sex

| PFAS | Boy (n=297) | | | |  | Girl (n=236) | | | |
| --- | --- | --- | --- | --- | --- | --- | --- | --- | --- |
|  | GM (GSD) | 25th | 50th | 75th |  | GM (GSD) | 25th | 50th | 75th |
| PFHxS | 2.7 (1.6) | 2 | 2.8 | 3.6 |  | 2.7 (1.5) | 2.1 | 2.6 | 3.4 |
| PFOS | 10.6 (1.7) | 7.5 | 10.4 | 15.1 |  | 11.1 (1.8) | 7.7 | 11.2 | 16.8 |
| PFOA | 19.6 (1.6) | 15 | 19.4 | 27.4 |  | 20.5 (1.5) | 15.8 | 20.5 | 27.3 |
| PFNA | 1.8 (1.6) | 1.3 | 1.7 | 2.4 |  | 1.9 (1.6) | 1.4 | 1.8 | 2.6 |
| PFDA | 2.0 (1.9) | 1.4 | 2.1 | 3.1 |  | 2.2 (2.0) | 1.5 | 2.1 | 3.4 |
| PFUdA | 1.5 (1.9) | 1 | 1.5 | 2.4 |  | 1.6 (2.0) | 1.1 | 1.7 | 2.5 |
| PFDoA | 0.1 (2.9) | 0.1 | 0.1 | 0.2 |  | 0.1 (2.8) | 0.1 | 0.1 | 0.2 |
| PFTrDA | 0.1 (2.9) | 0 | 0.1 | 0.2 |  | 0.1 (2.8) | 0.1 | 0.1 | 0.2 |

Note: GM, geometric mean; GSD, geometric standard deviation.

**Table S3** P-values of interaction item (PFASs*child sex) in the associations between maternal PFASs concentrations and child’s neurobehavioral problems assessed by ASQ at 4 years of age^*^.

| PFAS | Communication |  | Gross motor |  | Fine motor |  | Problem solving |  | Personal–social skills |
| --- | --- | --- | --- | --- | --- | --- | --- | --- | --- |
|  | p-value of interaction |  | p-value of interaction |  | p-value of interaction |  | p-value of interaction |  | p-value of interaction |
| PFHxS | 0.2189 |  | 0.8254 |  | 0.2204 |  | 0.7943 |  | 0.5965 |
| PFOS | 0.3500 |  | 0.3781 |  | 0.5209 |  | 0.0997^#^ |  | 0.0393^#^ |
| PFOA | 0.2551 |  | 0.0021^#^ |  | 0.6759 |  | 0.2726 |  | 0.0020^#^ |
| PFNA | 0.5162 |  | 0.7327 |  | 0.0731^#^ |  | 0.3258 |  | 0.2580 |
| PFDA | 0.4289 |  | 0.5285 |  | 0.2486 |  | 0.0279^#^ |  | 0.9210 |
| PFUdA | 0.4025 |  | 0.6364 |  | 0.3316 |  | 0.0330^#^ |  | 0.0620^#^ |
| PFDoA | 0.4222 |  | 0.7231 |  | 0.6333 |  | 0.5852 |  | 0.4500 |
| PFTrDA | 0.2906 |  | 0.8141 |  | 0.4203 |  | 0.8860 |  | 0.6252 |

^*^Using Poisson regression with robust variance estimates.

^#^ Statistically signiﬁcant differences (p < 0.10).

**Figure S1** Adjusted generalized additive model plots of ln-transformed PFAS concentrations with developmental problems in communication among offspring.


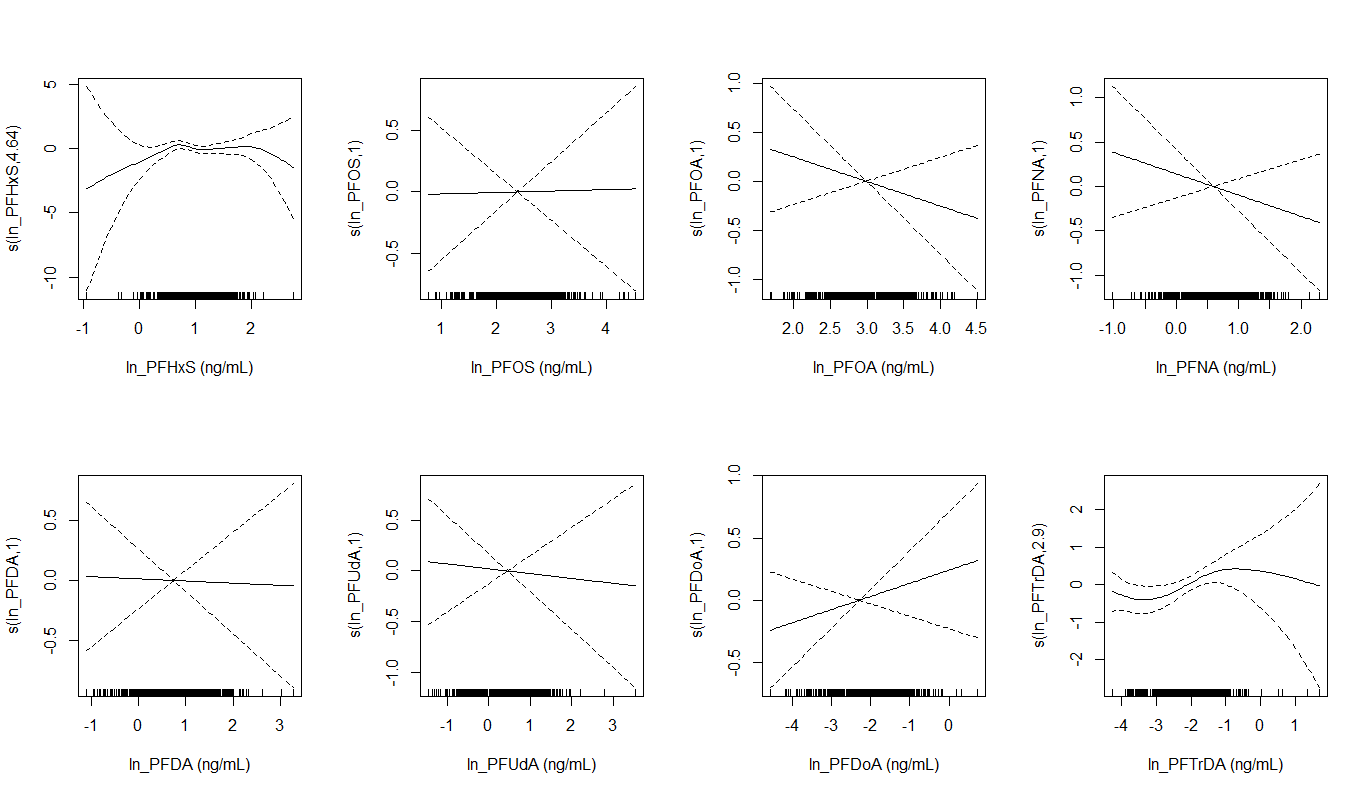


Notes: The middle line in each panel shows the estimated effect of maternal PFAS concentrations on developmental problems in communication (y-axis) plotted against the ln-transformed maternal PFAS concentrations (x-axis), after adjusting for covariates. Upper and lower lines show 95% confidence intervals of the estimated effect.

**Figure S2** Adjusted generalized additive model plots of ln-transformed PFAS concentrations with offspring developmental problems in Gross motor among offspring.


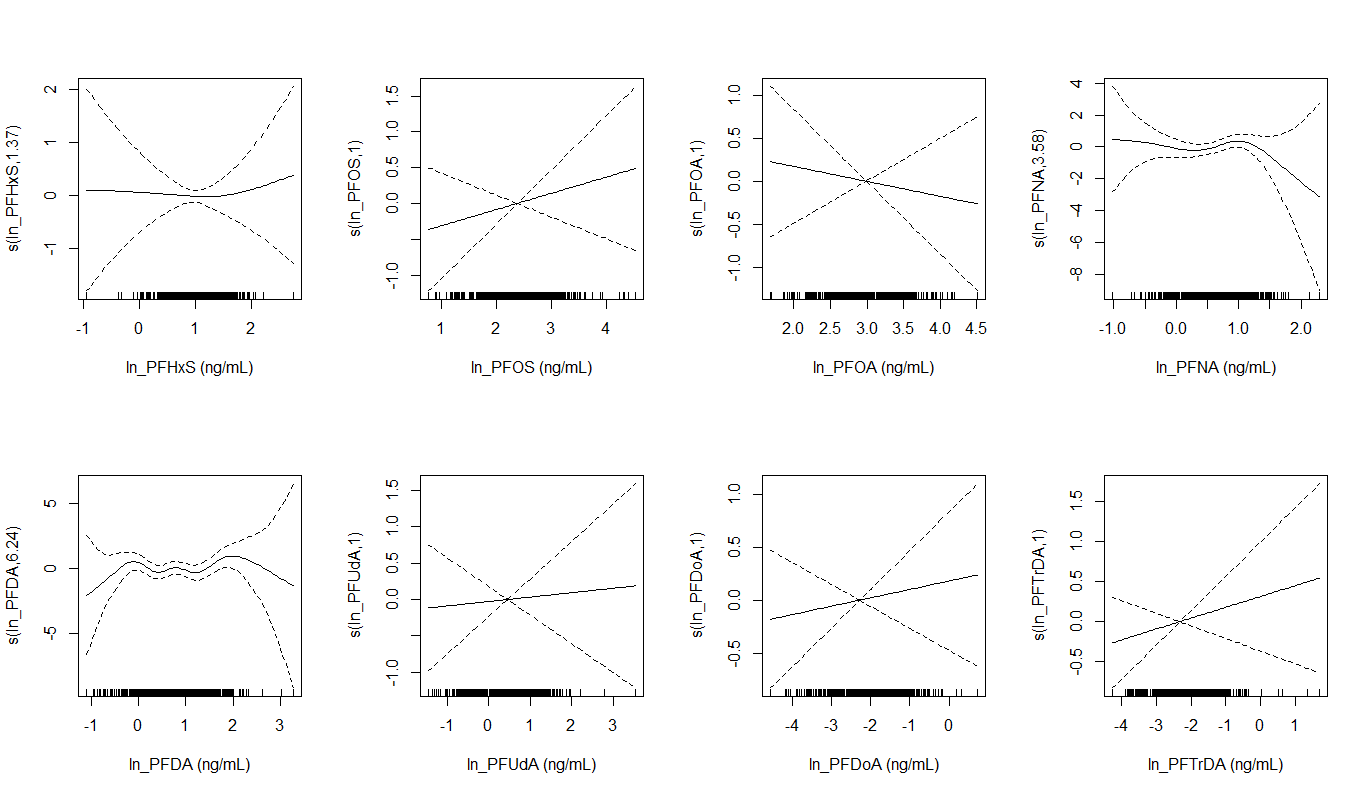


Notes: The middle line in each panel shows the estimated effect of maternal PFAS concentrations on developmental problems in Gross motor (y-axis) plotted against the ln-transformed maternal PFAS concentrations (x-axis), after adjusting for covariates. Upper and lower lines show 95% confidence intervals of the estimated effect.

**Figure S3** Adjusted generalized additive model plots of ln-transformed PFAS concentrations with developmental problems in Fine motor among offspring.


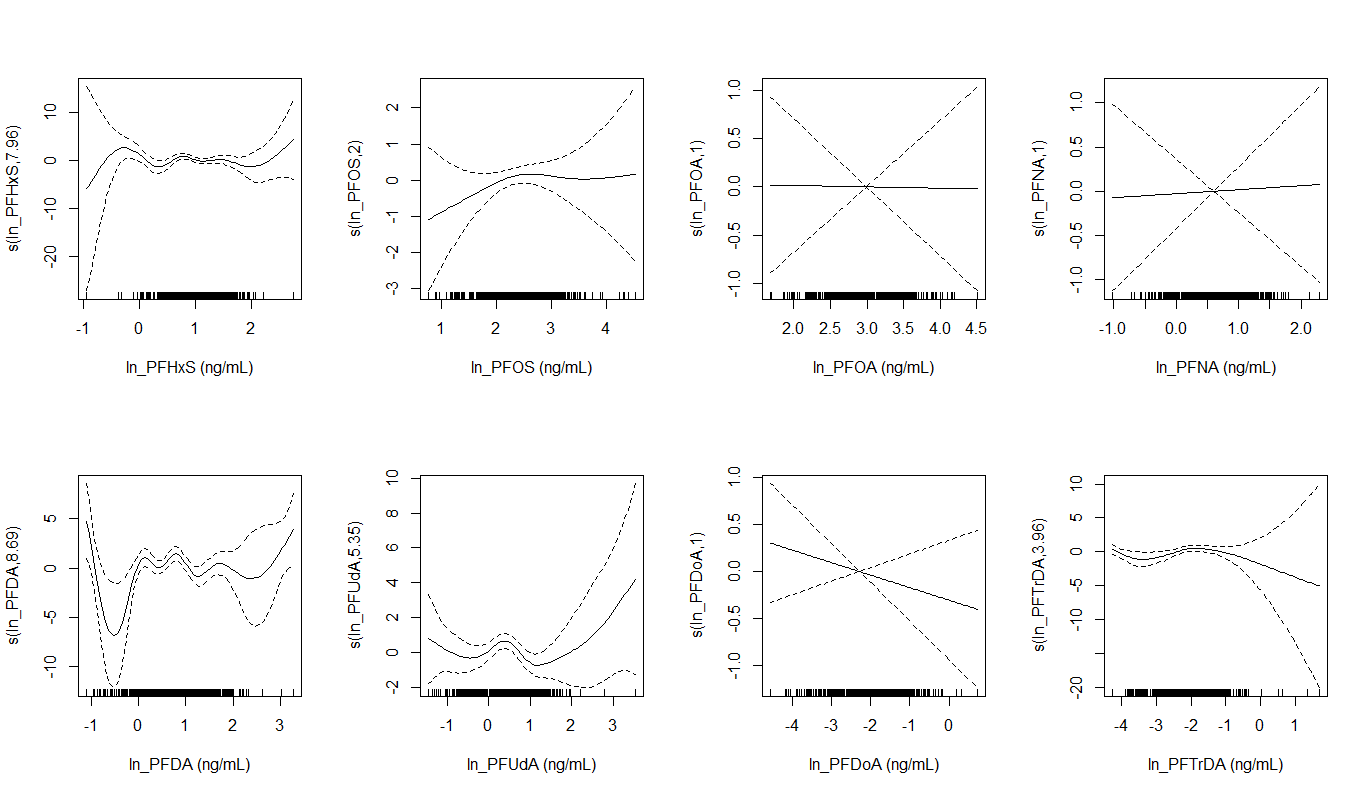


Notes: The middle line in each panel shows the estimated effect of maternal PFAS concentrations on developmental problems in Fine motor (y-axis) plotted against the ln-transformed maternal PFAS concentrations (x-axis), after adjusting for covariates. Upper and lower lines show 95% confidence intervals of the estimated effect.

**Figure S4** Adjusted generalized additive model plots of ln-transformed PFAS concentrations with developmental problems in Problem solving among offspring.


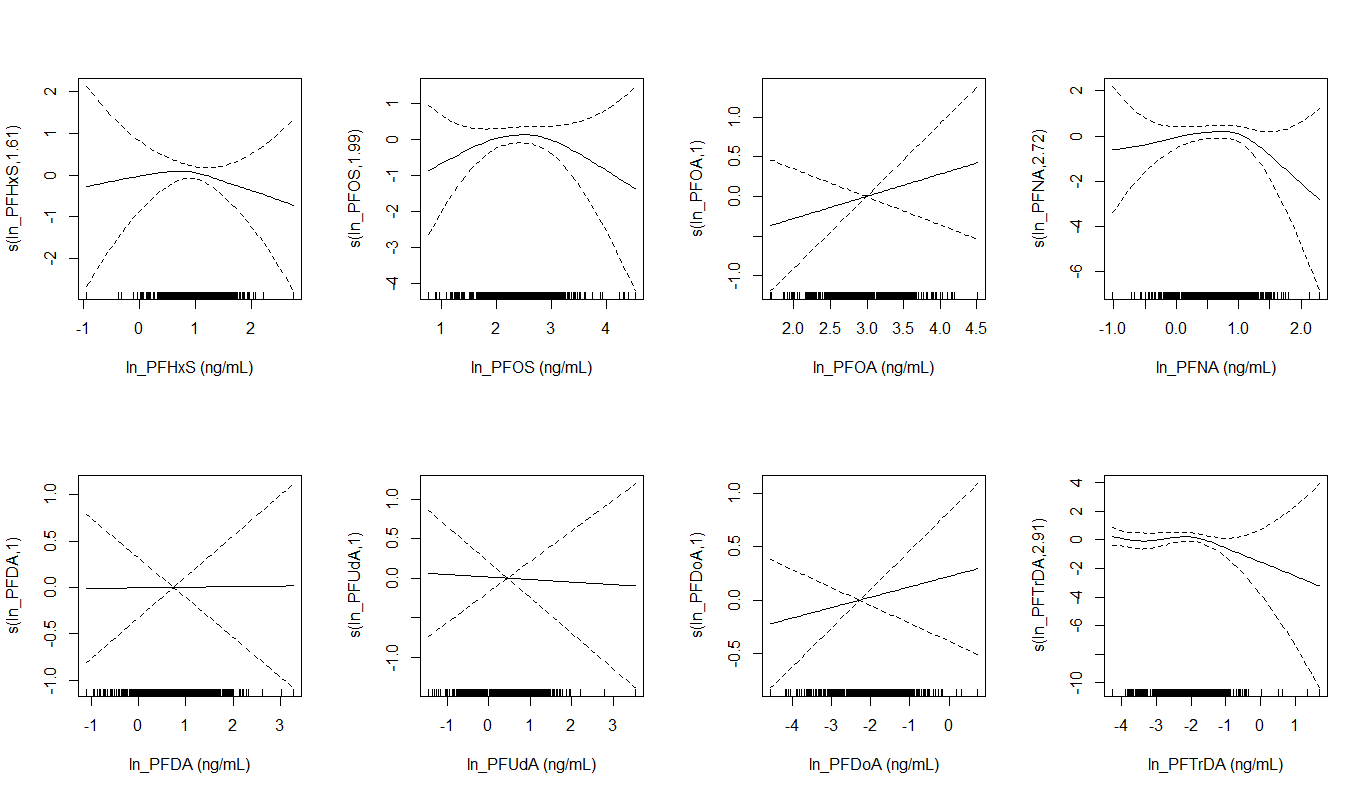


Notes: The middle line in each panel shows the estimated effect of maternal PFAS concentrations on developmental problems in Problem solving (y-axis) plotted against the ln-transformed maternal PFAS concentrations (x-axis), after adjusting for covariates. Upper and lower lines show 95% confidence intervals of the estimated effect.

**Figure S5** Adjusted generalized additive model plots of ln-transformed PFAS concentrations with developmental problems in Personal-social skills among offspring.


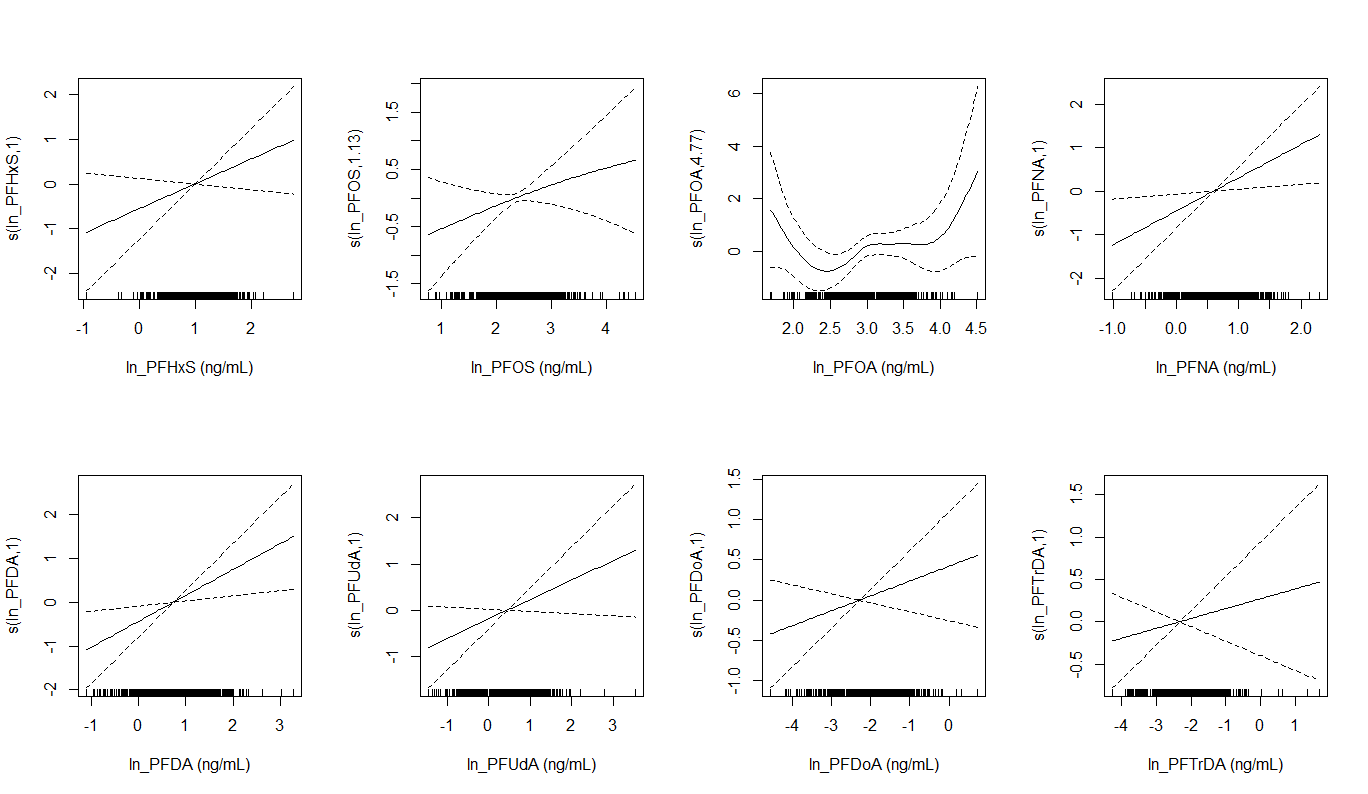


Notes: The middle line in each panel shows the estimated effect of maternal PFAS concentrations on developmental problems in Personal-social skills (y-axis) plotted against the ln-transformed maternal PFAS concentrations (x-axis), after adjusting for covariates. Upper and lower lines show 95% confidence intervals of the estimated effect.
